# ypmS promotes capsular polysaccharide shedding and in vivo fitness of Group B Streptococcus

**DOI:** 10.1099/mic.0.001763

**Published:** 2026-09-02

**Authors:** Molly E. Sharp, Sanjana Sankaran, Michelle J. Vaz, Sydney Haldeman, Emily Dembinski, Adam J. Ratner

**Affiliations:** 1 Department of Microbiology, New York University Grossman School of Medicine, New York, NY, USA; 2 Department of Pediatrics, New York University Grossman School of Medicine, New York, NY, USA

**Keywords:** capsule, colonization, *Streptococcus agalactiae* (Group B *Streptococcus*)

## Abstract

Group B *Streptococcus* (GBS) is a leading cause of neonatal sepsis and meningitis worldwide. Capsular polysaccharide (CPS) is a major virulence factor that aids GBS in colonizing the gastrointestinal (GI) and vaginal tracts, surviving in whole blood and evading the immune system. CPS is also the basis for a GBS CPS-protein conjugate vaccine candidate. In addition to its function on the surface of the bacteria, CPS can also be released and shed into the environment. In *Streptococcus pneumoniae*, shed CPS has been shown to absorb antimicrobial peptides and CPS-specific antibodies, allowing the bacteria to evade the innate immune system and potentially limiting vaccine protection. In this work, we show that CPS shedding occurs in ~75% of GBS isolates that we screened from a large library of clinical strains. We then identified *ypmS* as a genetic determinant of CPS shedding using an indexed transposon library. We used a clean in-frame deletion mutant of *ypmS* to assess the importance of shedding in GBS colonization and immune evasion. We explored the role of *ypmS* in colonization using murine models of GI and vaginal co-colonization and showed that the wild-type strain outcompeted the Δ*ypmS* mutant in both scenarios. We also assessed the effect of shed capsule on opsonophagocytosis. Free CPS was protective against bacterial killing in an opsonophagocytic killing assay using CPS-specific antibodies. With this work, we have identified a novel genetic cause of CPS shedding and shown its contribution to GBS colonization fitness and immune evasion.

## Introduction

Group B *Streptococcus* (GBS) colonizes the human gastrointestinal (GI) and vaginal tracts [1–3]. Vaginal colonization exposes infants to GBS during birth [4]. Invasive infection of the neonate can cause bacteraemia and meningitis resulting in lasting neurological impairment or death [5]. Current interventions include intrapartum antibiotic prophylaxis, which decreases early-onset disease (days 0–6 of life) but does not affect late-onset disease (LOD) (days 7–89 of life) [6–8]. GBS remains a leading cause of neonatal infection indicating the need for alternative preventative strategies, such as a vaccine [9–11].

GBS is an encapsulated bacterium. It has ten distinct serotypes (Ia, Ib, II, III, IV, V, VI, VII, VIII and IX) based on the unique monosaccharides and linkages that make up its capsular polysaccharide (CPS). Being the outermost component of the cell envelope, the capsule is the first point of contact in bacterial and host cell interactions and the first line of defence against the host immune system. Capsule promotes GBS colonization of the GI and vaginal tracts, ascending infection and survival in whole blood [12–16]. All known GBS CPS types contain sialic acid, which protects the bacteria from complement deposition and alternative pathway activation and interacts with human Siglec-9 to dampen the neutrophil response [17–20].

Some encapsulated bacteria shed CPS from their surface [21–25]. Free CPS from *Klebsiella pneumoniae*, *Streptococcus pneumoniae* (pneumococcus) and *Pseudomonas aeruginosa* was found to neutralize the bactericidal activity of antimicrobial peptides (APs) polymyxin B and human neutrophil α-defensin 1 by directly binding these APs [23]. The presence of APs also increased the amount of CPS that these bacteria shed [23]. In *S. pneumoniae*, LytA, an autolysin, is necessary for active CPS shedding in response to the AP LL-37 [21]. LytA cleaves peptidoglycan, causing CPS shedding and subsequent loss of surface-associated capsule but not death. The reduction in capsule resulted in increased survival of the pneumococcus in the presence of LL-37 and increased invasion into epithelial cells [21]. Because CPS is negatively charged, shedding CPS could be a strategy to absorb cationic APs and increase survival.

CPS shedding has also been implicated in serotype-specific efficacy differences for the 13-valent pneumococcal conjugate vaccine (PCV13). Serotype 3 sheds more CPS than other serotypes included in PCV13, likely due to its CPS being anchored in the membrane as opposed to being covalently bound to the peptidoglycan [22, 26]. In an opsonophagocytic killing (OPK) assay using serum from CPS-immunized rabbits, addition of serotype 3 CPS inhibited bacterial killing in a dose-dependent manner [22]. Shed CPS protected the bacteria from opsonophagocytosis by absorbing serotype-specific antibodies. As serotype 3 is one of the main causes of breakthrough cases of *S. pneumoniae* post-vaccination with PCV13 [27–29], this may be due to its increased shedding phenotype.

Overall, the literature shows CPS shedding can function as a defence mechanism against the host’s innate and adaptive immune response, adding to capsule’s role as a virulence factor. While CPS shedding has been observed in GBS, its mechanism and function have not been determined [30–32]. Here, we identify *ypmS* as a factor important for CPS shedding in GBS. We show that *ypmS* aids GBS in colonizing the murine vaginal and GI tracts and that shed CPS is a mediator of protection against OPK with anti-CPS antibodies.

## Results

### CPS shedding is the dominant phenotype in clinical isolates of GBS

**Fig. 1.**
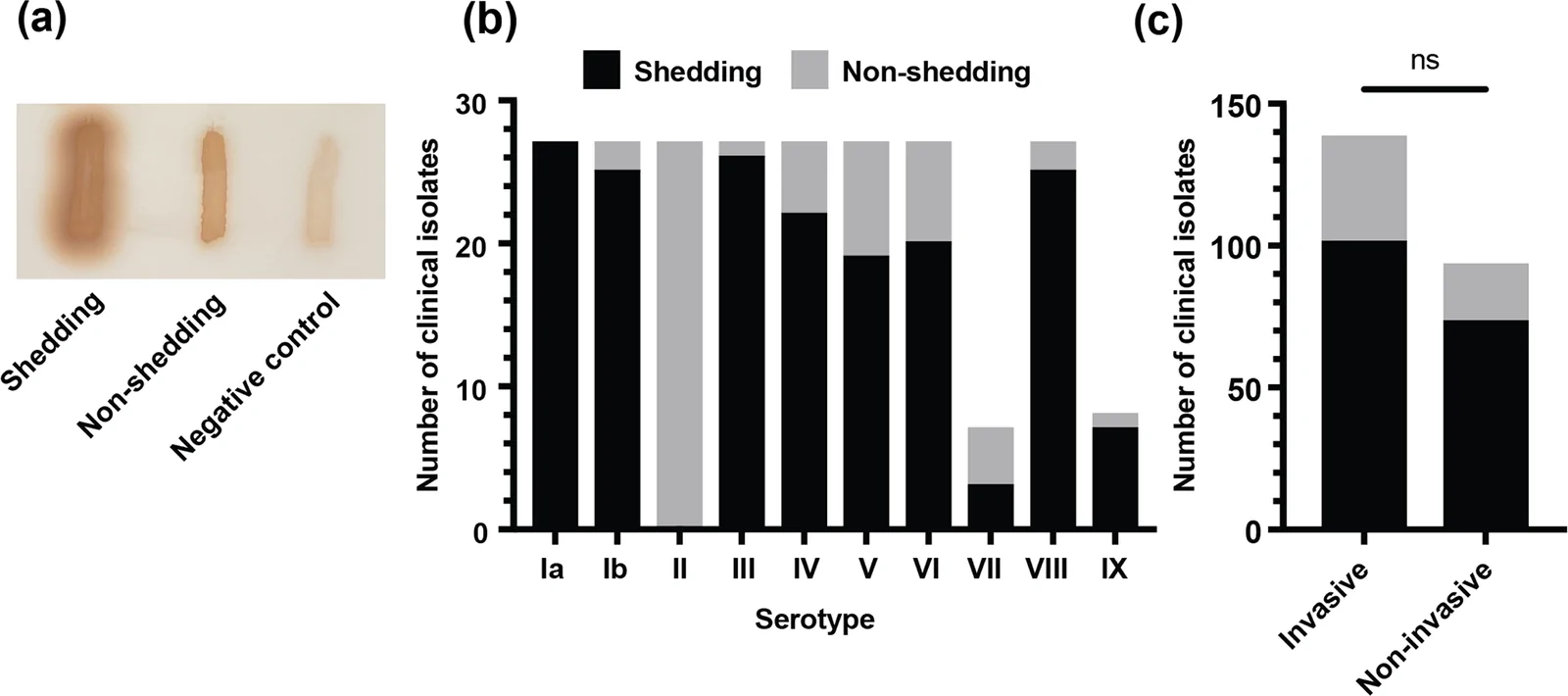
Prevalence of the shedding and non-shedding phenotypes in clinical isolates of GBS. Phenotype was determined by colony immunoblot using serotype-specific primary antibody. (**a**) Example immunoblot performed on patched colonies showing both phenotypes in comparison with a negative control. Shedding isolates have a halo around the patched colony. The halo is absent in non-shedding isolates. The negative control was a GBS isolate of a different serotype and was not recognized by the serotype-specific antibody. (**b**) Shedding vs non-shedding status broken down by serotype. (**c**) Shedding vs non-shedding status broken down by invasiveness. A χ^2^ test revealed no significant difference (ns) in the frequency of shedding and non-shedding phenotypes between invasive and non-invasive strains.

Although capsule shedding in GBS has been observed, the frequency of this phenotype in primary clinical isolates is unknown. We sought to determine the relative prevalence of shedding among GBS isolates that colonize and cause disease in humans. We evaluated a set of clinical isolates collected by the Centers for Disease Control and Prevention’s Active Bacterial Core surveillance and by our own group at New York University (NYU) Langone for the shedding phenotype. We screened isolates by patching individual colonies of each isolate onto tryptic soy (TS) agar and immunoblotting the patches using serotype-specific antibodies. Isolates were positive for shedding if they displayed a ‘halo’ around the patch, indicating that capsule had been released from the surface of the bacteria and diffused into the agar around the patch (Fig. 1a). Patches without a halo were classified as non-shedding isolates, although it should be noted that this classification is inclusive of isolates that express capsule and do not shed and those isolates that are acapsular. We screened a total of 231 isolates (27 for each serotype when possible, based on number of available isolates) and found that 174 isolates shed capsule (75.3%), whereas 57 did not shed capsule (24.7%) (Fig. 1b). Most serotypes showed a mixture of shedding and non-shedding strains, except serotype Ia and II. All tested serotype Ia isolates shed capsule, whereas all serotype II isolates did not shed capsule. We were able to screen only a small number of serotype VII and IX isolates because these serotypes are relatively rare in the population. Serotype VIII is also rare, but we had many more strains in our collection from a serotype VIII study we had previously conducted [33]. We also evaluated colonizing vs invasive status of the isolates. Shedding was seen in 73.2% of the invasive isolates and in 78.5% of the non-invasive isolates, indicating no relationship between shedding phenotype and isolate invasiveness (*P*>0.05 by χ^2^ test; Fig. 1c). Overall, CPS shedding is the dominant phenotype in clinical isolates of GBS with the notable exception of the tested serotype II strains, which did not shed capsule.

### *ypmS* is a genetic determinant of CPS shedding

To investigate this capsule shedding phenotype, we screened a collection of transposon insertion mutants to identify potential genetic determinants of shedding. We used a previously described indexed transposon library containing 1,920 individual mutants with unique transposon insertions throughout the A909, serotype Ia genome [34]. Each mutant was spotted onto TS agar, and spots were immunoblotted with serotype Ia-specific antibody, as described for the clinical isolate screen above. Mutants with reduced or absent halos were identified potentially to contain insertions in genes essential for shedding. From this initial screen, we identified 28 transposon mutants with an altered shedding phenotype. However, this method could not distinguish between mutants deficient in capsule production and mutants that could make capsule but not shed it. To differentiate these factors among our initial hits, we performed enzyme-linked lectin assays (ELLAs) using an α2,3-linked sialic acid-specific lectin to assess the presence of capsule on the bacterial surface and dot blot assays using a serotype Ia-specific antibody to probe the supernatant for shed capsule. When compared to wild-type A909 and an acapsular control, Δ*cpsE,* a single mutant, Tn848, both retained a substantial amount of surface-associated capsule and had reduced capsule present in the supernatant (Fig. S1, available in the online Supplementary Material), indicating a specific deficiency in CPS shedding.

**Fig. 2.**
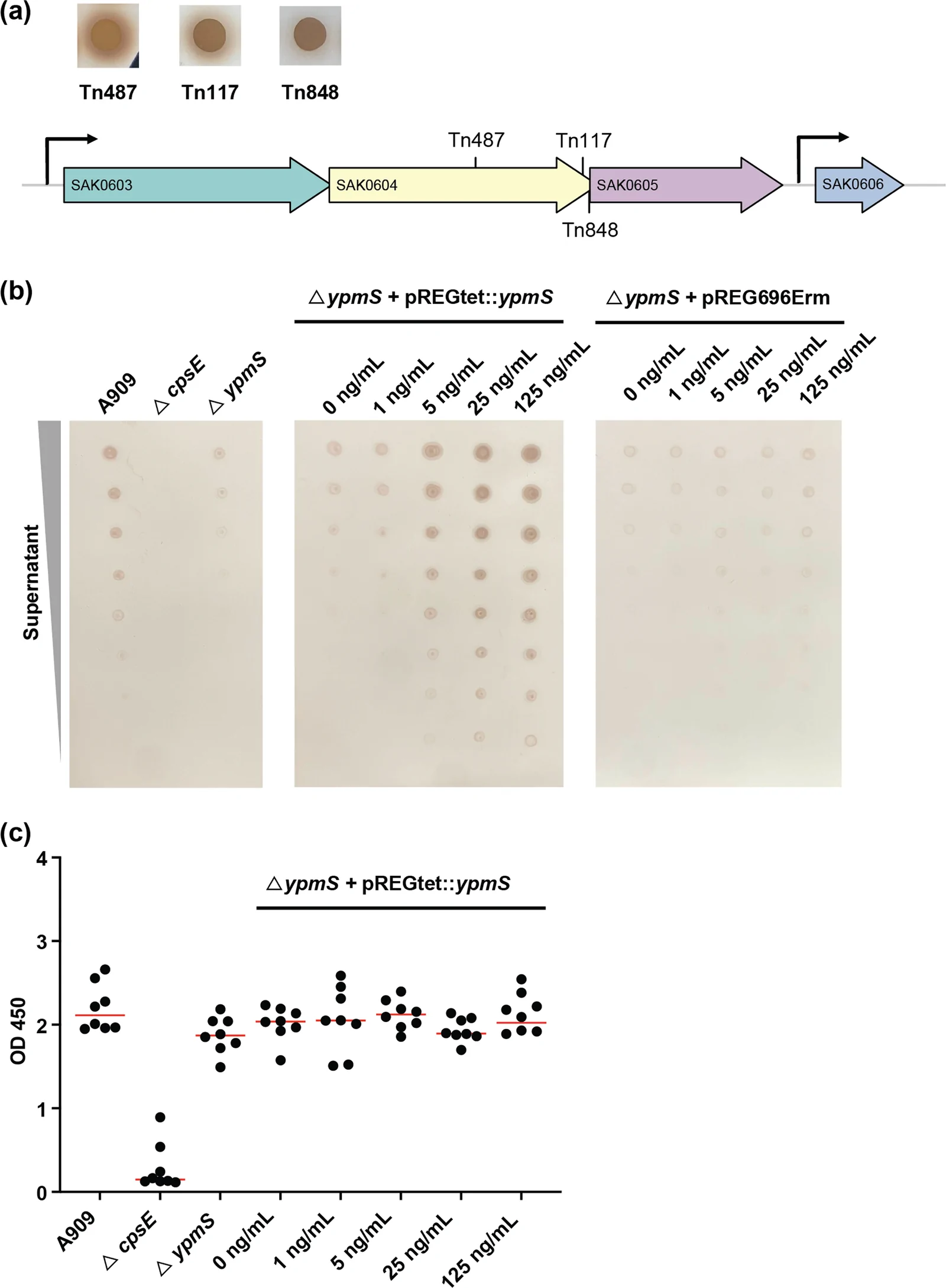
Identification of *ypmS* as a genetic determinant of CPS shedding. (**a**) Tn848 was identified in a screen of an A909 indexed transposon library to have a non-shedding phenotype. The location of all three Tn mutants located in SAK0604 is shown along with their shedding phenotype. (**b**) Dot blots of 1 : 2 dilutions of supernatant (decreasing concentrations indicated by the grey wedge) using a serotype Ia-specific antibody to detect shed CPS. Concentrations of aTC for induction of Δ*ypmS*+pREGtet::*ypmS* or Δ*ypmS*+pREG696Erm are indicated above the blots. (**c**) Whole-cell ELLA using MAL I to assess surface-associated CPS. ANOVA with Tukey’s multiple comparisons test was used to assess significance. No statistical difference was found between A909, Δ*ypmS*, or any of the induction conditions for Δ*ypmS*+pREGtet::*ypmS*. Δ*cpsE* was significantly different from all other strains (*P*<0.0001). The concentration of aTC used for each induction is displayed on the x-axis. Data from (**b**) and (**c**) were generated from the same culture grown to an OD_600_ of 0.6 and split into (**b**) a supernatant fraction and (**c**) a pellet fraction.

The transposon insertion in mutant Tn848 was located at the 3′ end of the gene SAK0604 and just one nt upstream of the start codon of the next gene, annotated as encoding a YpmS family protein (Fig. 2a). We noted that the transposon library contained two other mutants in the SAK0604 gene, but both shed capsule (Fig. 2a). The location of the transposon insertion, as well as the phenotype of the other mutants in this gene, suggested that the non-shedding phenotype might be due to a polar effect from the transposon insertion on the downstream gene, *ypmS*. The indexed library did not contain any mutants with a transposon insertion in *ypmS*, so we created clean, in-frame deletions of both genes to confirm the effect of each on the shedding phenotype. The deletion mutant ΔSAK0604 retained surface-associated CPS (although it and Tn848 both had less capsule than wild-type A909) and had little observable decrease in the amount of shed CPS detected in the supernatant (Fig. S1). Δ*ypmS* on the other hand, maintained wild-type levels of surface-associated capsule and displayed a marked decrease in shed capsule detected in the supernatant (Fig. 2b–c). It is unclear why we observed a disparity in surface-associated CPS for ΔSAK0604 and Tn848, but the difference in shed CPS indicated *ypmS*, not SAK0604, was responsible for the shedding phenotype. To validate *ypmS* as a potential genetic determinant of capsule shedding, we complemented *ypmS* on a plasmid under the control of an anhydrotetracycline (aTC) inducible promoter (pREGtet::*ypmS*). When Δ*ypmS*+pREGtet::*ypmS* was induced with increasing concentrations of aTC, we saw a dose-dependent increase of CPS in the supernatant (Fig. 2b). To ensure that this finding was the result of *ypmS* expression and not a non-specific effect of aTC exposure, we also exposed the Δ*ypmS*+pREG696 Erm empty vector control to the same aTC concentrations and saw no increase in CPS shed into the supernatant (Fig. 2b).

**Fig. 3.**
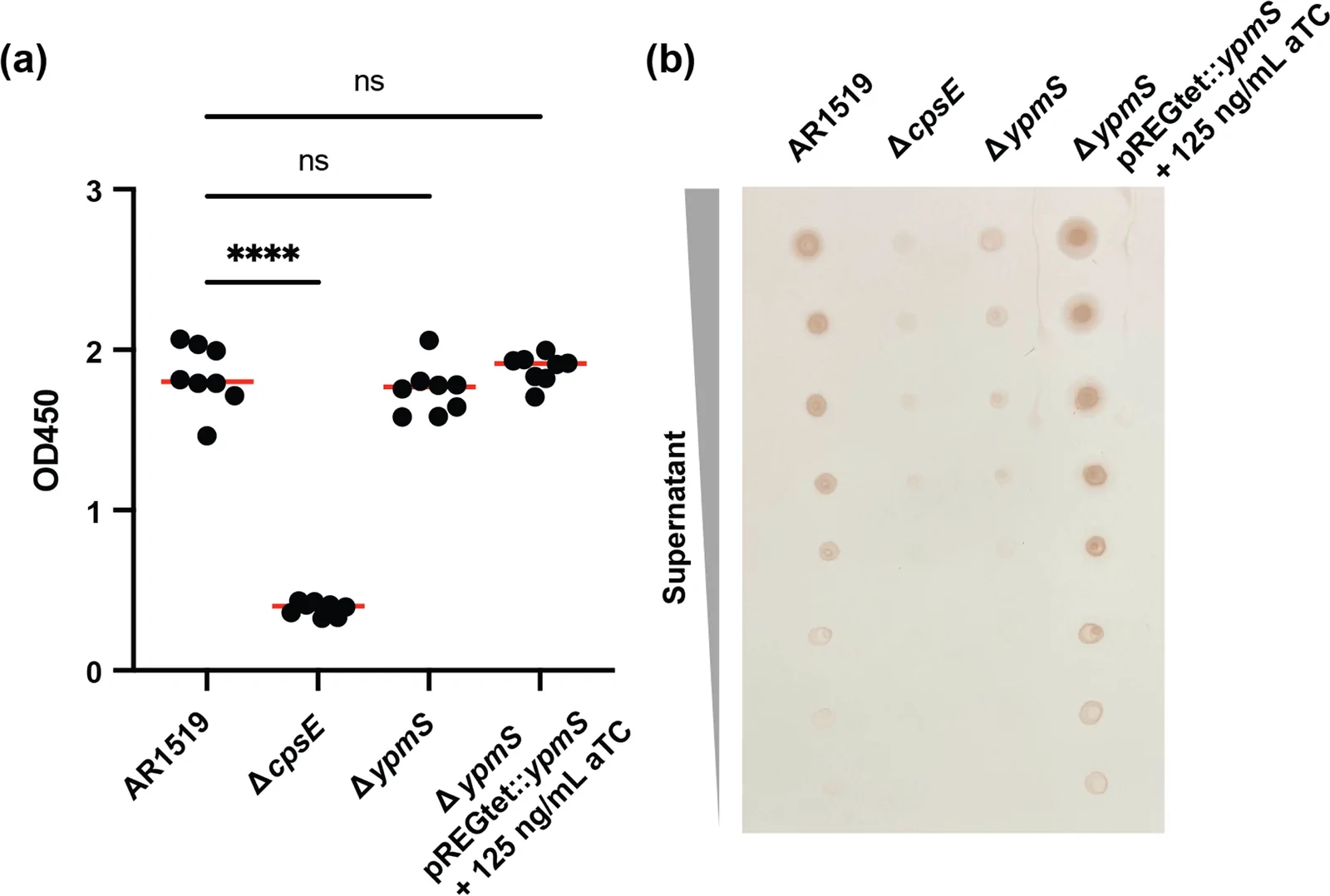
Confirmation of Δ*ypmS* non-shedding phenotype in a serotype V background. A concentration of 125 ng ml^−1^ of aTC was used to induce *ypmS* expression in the complemented mutant. (**a**) Whole-cell ELLA using MAL I to assess surface-associated CPS. (**b**) Dot blot of 1 : 2 dilutions of supernatant using a serotype V-specific antibody to detect shed CPS. Data from (**a**) and (**b**) were generated from the same culture grown to an OD_600_ of 0.6 and split into (**a**) a pellet fraction and (**b**) a supernatant fraction. Statistical significance was determined using ANOVA with Tukey’s multiple comparisons test (****P<0.0001).

To understand if this phenotype was strain- or serotype-specific, we also deleted *ypmS* in a serotype V GBS strain, AR1519. When compared to the wild-type strain and an acapsular control in the same genetic background, the Δ*ypmS* mutant had decreased CPS in the supernatant and maintained surface-associated capsule (Fig. 3a–b). The serotype V Δ*ypmS* mutant was also complemented with the same aTC-inducible plasmid pREGtet::*ypmS*. This complemented strain had increased CPS in the supernatant when induced with 125 ng ml^−1^ aTC, and it maintained wild-type levels of surface-associated capsule (Fig. 3a–b). Therefore, *ypmS* is responsible for GBS CPS shedding in multiple serotype backgrounds.

### *ypmS* promotes fitness in murine co-colonization

**Fig. 4.**
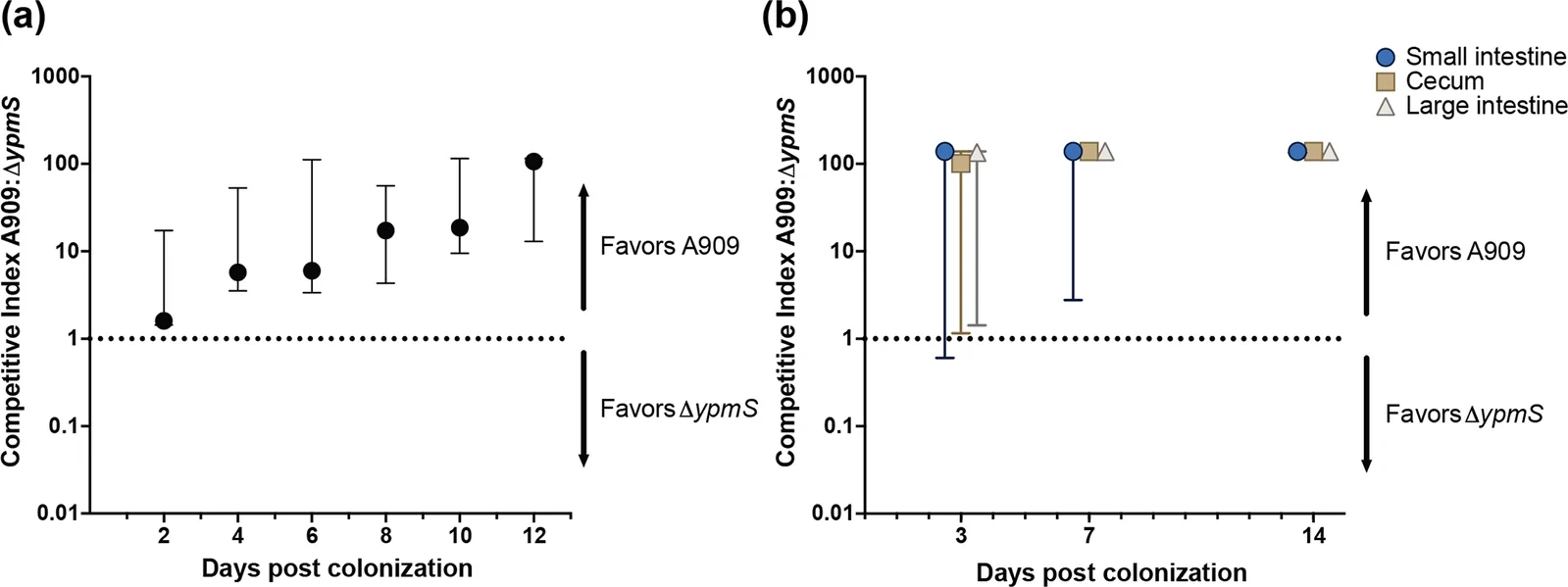
Wild-type A909 outcompetes the isogenic mutant Δ*ypmS* in murine models of (**a**) adult vaginal and (**b**) postnatal GI co-colonization. (**a**) One cohort of adult C57BL/6 J mice was vaginally colonized with equal amounts of A909 and Δ*ypmS* (*n*=5). Samples were collected by vaginally swabbing the mice on days 2, 4, 6, 8, 10 and 12 post-colonization. Swab contents were plated on CHROMagar. Colonies were patched onto TS and differentiated by colony PCR (40 colonies per swab). Data points represent median competitive indices, and error bars represent 95% CI. (**b**) Preweaning C57BL/6 J mice (12–14 days) were orally inoculated with equal amounts of A909 and Δ*ypmS* (in one experiment with three cohorts: day 3, n=6; day 7, n=7; and day 14, n=5). Small intestine, caecum and large intestine were harvested on days 3, 7 and 14 post-colonization. Homogenized organ samples were plated on CHROMagar. Colonies were patched onto TS agar and differentiated by colony PCR (40 colonies per organ, per mouse). Data points represent median competitive indices, and error bars represent 95% CI.

GBS colonization of the vaginal tract is a precursor to neonatal invasive infection [4]. Fitness in this setting requires establishing a niche and outcompeting other bacteria, including other GBS strains that may populate the vaginal tract simultaneously [35]. We hypothesized that GBS strains that shed capsule may have an advantage in establishing colonization by more effectively evading the immune system. To investigate the role of *ypmS* in colonization, we competed wild-type A909 and Δ*ypmS* in a murine model of vaginal co-colonization. On day 2 post-colonization, wild-type and Δ*ypmS* were both recovered, with a competitive index of 1.59 indicating a near equal ratio of wild-type to mutant. As colonization progressed, the wild-type strain began to outcompete Δ*ypmS,* and the competitive index increased to 5.75, 5.98, 17.25, 18.69 and finally 106.38 on days 4, 6, 8, 10 and 12 post-colonization, respectively (Fig. 4a). At each time point, at least one of the mice in the cohort had Δ*ypmS* recovered, but at all time points and for all mice, the majority of the recovered bacteria were wild-type. This indicates that *ypmS* is not necessary for colonization, but it is needed in a co-colonization environment to establish and sustain vaginal colonization in the presence of other GBS strains.

Colonization of the neonatal GI tract is considered a critical precursor to LOD [36]. We assessed the role of Δ*ypmS* in a murine model of GI co-colonization. Pups were colonized with equal amounts of wild-type A909 and Δ*ypmS* between days 12 and 14 of life. At days 3, 5 and 7 post-colonization, the median competitive index ranged from 100 to 139 across all recovered GI sites, including the small intestine, caecum and large intestine. On day 3, one mouse had near equal recovery of the wild-type and Δ*ypmS* strains, whereas all other mice had majority wild-type bacteria recovered (Fig. 4b). By day 7, Δ*ypmS* was detected in the small intestine of only one mouse, and the wild-type was the only bacterial strain recovered in all other animals (Fig. 4b). On day 14, the wild-type was the only bacterium recovered from all GI sites in the cohort (Fig. 4b). These findings suggest that *ypmS* enhances competitive fitness in murine gut colonization, suggesting a potential role in the development of strain dominance and consequently the emergence of GBS strains that could progress to neonatal disease.

### *ypmS* and shed CPS are protective against opsonophagocytosis

**Fig. 5.**
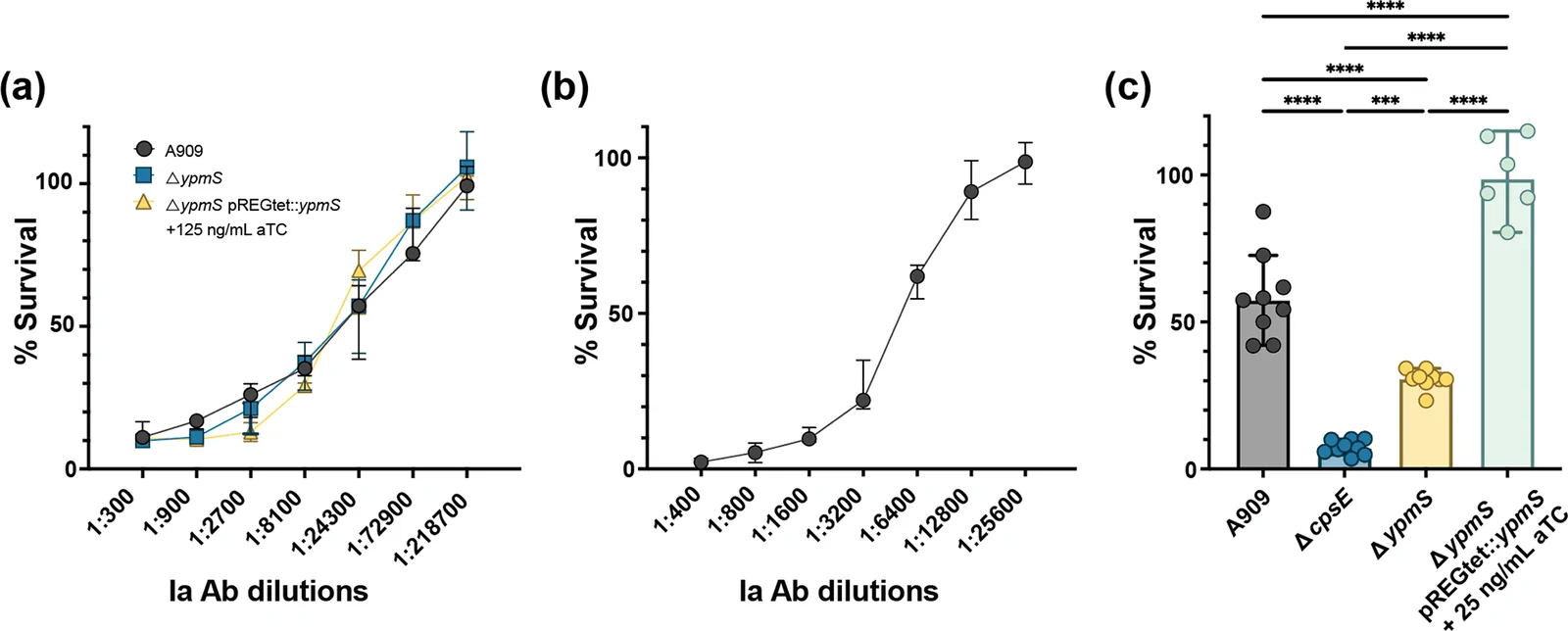
OPK of GBS is reduced by the addition of supernatant containing shed CPS. (**a**) Percent survival of the listed strains across a range of dilutions of serotype Ia-specific antibody. The complemented strain was induced with 125 ng ml^−1^ of aTC. Standard OPK conditions were performed, in which the bacteria and antibody were diluted in buffer and incubated for 30 min before differentiated HL-60 cells were added. Data points represent median and 95% CI from biological triplicates. (**b**) Percent survival curve of Δ*ypmS*+pREG696 Erm. The initial incubation of bacteria and antibody was performed in the presence of supernatant collected from Δ*cpsE* mutants instead of buffer. From this curve, a concentration of antibody that killed 90–95% of bacteria in the presence of supernatant was chosen for (**c**). Data points represent median and 95% CI from biological triplicates. (**c**) Difference in percent survival of Δ*ypmS*+pREG696 Erm in the presence of supernatant from A909, Δ*cpsE*, Δ*ypmS* and Δ*ypmS*+pREGtet::*ypmS* induced with 25 ng ml^−1^ aTC. All assays were performed with Ia antibody at a dilution of 1 : 800. Individual values represent biological replicates, and error bars represent the median and 95% CI. ANOVA with Tukey’s multiple comparisons test was used to compare supernatant conditions (***P<0.001, ****P<0.0001).

To investigate potential ramifications of shed CPS on a CPS-based vaccine, we performed an OPK assay using serotype Ia-specific antibody. We observed similar survival curves of A909, Δ*ypmS* and Δ*ypmS* +pREGtet::*ypmS* induced with 125 ng ml^−1^ of aTC under standard assay conditions (Fig. 5a). Under these conditions, the bacteria and antibody are incubated for 30 min in buffer to allow for opsonization. We theorized that this method allowed opsonization of the bacteria before CPS could be shed, and, because all the strains are equally encapsulated, surface CPS was what determined survival under standard assay conditions. Therefore, we were not detecting any effect that shed CPS may have on opsonization. We then repeated the assay but preincubated the bacteria and antibody with bacterial supernatant. We first determined a concentration of antibody that killed 90–95% of the bacteria in the presence of Δ*cpsE* supernatant to control for any non-specific effect of the supernatant on the assay (Fig. 5b). For all assays using supernatant, the empty vector control, Δ*ypmS*+pREG696 Erm was used as the base strain. The OPK assay was performed using a serotype Ia-specific antibody at a 1 : 800 dilution with addition of supernatant from A909, Δ*cpsE*, Δ*ypmS* or Δ*ypmS*+pREGtet::*ypmS* induced with 25 ng ml^−1^ aTC. In the presence of Δ*cpsE* supernatant, the median survival was 7.07%. The addition of Δ*ypmS* supernatant increased median survival to 30.64%. The addition of A909 supernatant increased median survival to 57.37%. Lastly, the addition of Δ*ypmS* +pREGtet::*ypmS* supernatant increased median survival to 98.675% (Fig. 5c). Taken together, these data show that increased quantities of free CPS in the bacterial environment enhance resistance to opsonization by serotype-specific antibodies and reduce bacterial killing.

## Discussion

Capsule has most rigorously been studied as a component of the cell envelope, but it may also be released from the bacterial surface and shed into the environment. This phenomenon has been observed in many encapsulated bacterial species including GBS, *S. pneumoniae, K. pneumoniae, P. aeruginosa, Streptococcus pyogenes* and *Acinetobacter baumannii* [21–25, 30–32]. While previous work observed CPS shedding in GBS, we report the first dedicated investigation into genetic factors important for CPS shedding and the impact of CPS shedding on GBS colonization and immune evasion.

Work by Kietzman *et al*. showed that *S. pneumoniae* actively shed its capsule in response to the presence of APs, suggesting shedding allows the bacteria to remodel its cell surface in response to its environment [21]. The mechanism *S. pneumoniae* uses to shed CPS is driven by LytA, an *N*-acetylmuramoyl-l-alanine amidase and autolysin responsible for cell wall cleavage [37]. LytA can be induced by the AP LL-37. It cleaves the peptidoglycan in a spatially distinct area from the site of autolysis cleavage, causing CPS shedding and subsequent loss of surface-associated capsule but not death. The reduction in capsule resulted in increased survival of the pneumococcus in the presence of LL-37 and increased invasion into epithelial cells [21]. The authors speculated that these findings suggest dynamic encapsulation of the pneumococcus depending on its environment and stimuli.

GBS does not have a *lytA* homologue or an autolysin similar to *S. pneumoniae*. Instead, we have shown that *ypmS* is required for CPS shedding in GBS. Under standard growth conditions, GBS sheds CPS but maintains a consistent amount of capsule on its surface. In the Δ*ypmS* and complemented mutants, we see that regardless of shedding phenotype, surface-associated capsule remains constant. Therefore, we do not currently have evidence to suggest that GBS capsule shedding is responsive to environmental signals. Instead, shedding may be due to an overproduction of capsule. In a nutrient-rich environment such as TS broth, more capsule than can physically be sustained on the cell surface may be produced, causing shedding. In this case, we would hypothesize that *ypmS* would affect the total amount of capsule produced rather than controlling shedding directly.

When we competed wild-type A909 and Δ*ypmS* in murine models of adult vaginal and postnatal GI colonization, wild-type A909 outcompeted Δ*ypmS*, indicating a role for *ypmS* in *in vivo* polymicrobial settings. Shedding CPS could provide an advantage to the wild-type strain in these models by aiding in immune evasion or by reducing surface-associated capsule at the epithelial surface, allowing the bacteria to adhere to cells and more quickly establish a niche. Encapsulation inversely correlates with adherence and invasion of GBS *in vitro* [38, 39]. As previously discussed, we do not currently have evidence that GBS reduces its surface-associated CPS *in vitro* or *in vivo* like pneumococcus. GBS employs a different shedding mechanism because YpmS is not an autolysin like LytA and therefore may not be able to directly remove surface-associated CPS. Alternatively, if shedding is able to remove surface-associated CPS, we have yet to identify any environmental stimuli that could trigger GBS shedding similar to the pneumococcal response to the AP LL-37. If GBS encapsulation is dynamic, it may only be observable *in vivo*, which could explain both the colonization results and the high prevalence of shedding strains in our clinical isolate library. It would be valuable to assess if GBS similarly increases shed capsule and reduces surface-associated capsule in response to the presence of APs.

In the *in vivo*, polymicrobial environment, the proximity of GBS strains may also contribute to the effect of shedding on competition. We do not know how GBS strains are spatially segregated in the vaginal or GI tracts when multiple strains of GBS concurrently colonize these environments. We also do not know how far shed CPS disseminates from the bacteria once it is released. Understanding these two factors could inform why we do not see protection of the non-shedding strain by the released CPS from the shedding strain in our *in vivo* competition experiments.

The clinical isolate screen we performed demonstrated the consistency of the shedding phenotype in invasive and colonizing strains of GBS. Among the non-shedding strains, we observed a range of reactivity among patches, some being comparable to shedding patches in intensity, likely indicating encapsulation without shedding, while other patches exhibited a lesser intensity, potentially due to a change in capsule expression. This screen also revealed an unexpected difference in shedding phenotypes between serotypes. Specifically, serotype II was the only serotype in which the strains tested did not shed capsule. The non-shedding phenotype of serotype II strains could be due to the way the CPS is attached to the surface of the bacteria, the structure of the CPS itself or a difference in regulation. The linkage between CPS and the surface of GBS has only been defined for the serotype III strain COH1 [40]. Serotype II CPS could be attached in a different way, rendering it less susceptible to shedding. The serotype II CPS repeating unit is the only CPS with a pentasaccharide backbone and two monosaccharide side chains (one being sialic acid), which could affect capsule density and packing, making it more difficult for the CPS to be disrupted and shed. The non-shedding phenotype of serotype II strains remains a question for further investigation.

CPS-protein conjugate vaccines have proven effective against other bacterial pathogens such as *Haemophilus influenzae* type b, *Neisseria meningitidis* and *S. pneumoniae* [41]. The vaccine that is furthest along in clinical trials for GBS is a polysaccharide protein-conjugate vaccine covering the six most common disease-causing serotypes (Ia, Ib–V) [42]. Shed capsule may absorb serotype-specific antibodies, potentially decreasing the protection from a CPS-based vaccine [22]. *S. pneumoniae* serotype 3 capsule is not covalently bound to peptidoglycan the way most other serotypes are; instead, it is anchored in the membrane [26]. Choi *et al*. showed that serotype 3 shed more capsule into the supernatant than covalently bound capsule types [22]. In an OPK assay using serotype 3-specific rabbit serum, addition of serotype 3 CPS inhibited bacterial killing in a dose-dependent manner [22]. Additionally, as little as 0.2 µl of serotype 3 supernatant abolished passive protection with anti-serotype 3 antibodies in a murine model of intraperitoneal sepsis in comparison to 25 µl of supernatant from a covalently anchored strain [22]. Shed capsule may prevent complement-mediated phagocytosis by absorbing capsular antibodies and allowing the bacteria to escape recognition by phagocytes. We tested the effect of shed CPS on opsonization with CPS-specific antibodies to understand potential interactions with antibodies produced by a CPS-based vaccine. We found that supernatants with higher amounts of free CPS were more protective against OPK, suggesting potential for a similar effect of GBS CPS shedding on vaccine effectiveness.

YpmS was an unexpected mediator of CPS shedding. It is an uncharacterized protein that contains no conserved domains with enzymatic function. The structure of YpmS, as predicted by AlphaFold [43, 44], has a transmembrane α-helix domain at the N-terminus, followed by a predicted extracellular domain. Basic local alignment search tool (blastp) was used to search the National Center for Biotechnology Information’s non-redundant protein sequences (nr) database for YpmS orthologues. This search returned other YpmS family proteins in a wide range of streptococcal species and other *Firmicutes*, suggesting that *ypmS* may have a conserved function beyond GBS.

In conclusion, we have shown that CPS shedding occurs in most clinical isolates of GBS and that free CPS is protective against opsonization with anti-CPS antibodies and subsequent phagocytic killing. The mechanism by which shedding occurs in GBS remains to be elucidated, but we have identified *ypmS* as a genetic determinant of this phenotype, providing a foundation for future work. Understanding CPS shedding in GBS may be useful in predicting CPS vaccine effectiveness.

## Methods

### Bacterial strains and growth conditions

Bacterial strains used in this work are listed in Table 1. GBS strains were grown stationary at 37 °C in TS broth. *Escherichia coli* strains were grown shaking at 37 °C in Luria–Bertani broth. All plasmids in this study besides pREG696 are erythromycin-resistant, so plasmid maintenance was achieved with 300 µg ml^−1^ of erythromycin in *E. coli* strains and 5 µg ml^−1^ of erythromycin in GBS strains. The *E. coli* strain containing pREG696 was maintained with 150 µg ml^−1^ of spectinomycin. Strains with pMBSacB were grown at 28 °C, as it is temperature sensitive. The *Himar1* transposon is erythromycin resistant, so the transposon library was also grown in the presence of 5 µg ml^−1^ of erythromycin. Wild-type and in-frame deletion strains of GBS did not require antibiotic supplementation.

**Table 1.** GBS strains and plasmids used in this study

Strain/plasmid	Description	Reference/source
5-α competent *E. coli*	Chemically competent cloning strain	New England Biolabs item no. C2987H
A909	Serotype Ia used as the genetic background for all type Ia mutants made in this study	[54]
Δ*cpsE*	A909 with an in-frame deletion of *cpsE* from start to stop codon of *cpsE*; acapsular control	[14]
Tn848	A909 *Himar1* transposon mutant; the transposon is inserted at the end of the gene SAK0604	[34]
Δ*ypmS*	A909 with an in-frame deletion of *ypmS* from stop codon of SAK0604 to stop codon of *ypmS*	This study
Δ*ypmS*+pREGtet::*ypmS*	Δ*ypmS* complemented with *ypmS* under the control of an aTC inducible promoter	This study
ΔSAK0604	A909 with an in-frame deletion of SAK0604 from stop codon of SAK0603 to start codon of *ypmS*	This study
ΔSAK0604 revertant	A909 revertant control for allelic exchange mutagenesis of ΔSAK0604	This study
AR1519	Serotype V used as the genetic background for all type V mutants made in this study	[55]
Δ*cpsE*	AR1519 with an in-frame deletion of *cpsE* from start to stop codon of *cpsE*; acapsular control	This study
Δ*ypmS*	AR1519 with an in-frame deletion of *ypmS* from stop codon of SAK0604 to stop codon of *ypmS*	This study
Δ*ypmS*+pREGtet::*ypmS*	AR1519Δ*ypmS* complemented with *ypmS* under the control of an aTC inducible promoter	This study
pMBSacB	Temperature-sensitive, erythromycin-resistant plasmid containing p23-*sacB* for sucrose-counterselectable GBS mutagenesis	[45]
pGBSedit	Shuttle vector for CRISPR/Cas12a GBS mutagenesis; this plasmid also contains the P_xyl/tet_ inducible promoter and TetR repressor used to construct pREGtet::*ypmS*	[46]
pREG696	Low copy plasmid with *axe-txe* module for stable plasmid complementation; spectinomycin resistant	[48]
pOri23	Shuttle vector containing the erythromycin resistance cassette used to construct pREG696Erm	[49]
pREG696Erm	pREG696 with the spectinomycin resistance cassette replaced with an erythromycin resistance cassette	This study
pREGtet::*ypmS*	pREG696Erm with the addition of *ypmS* under the control of the aTC inducible promoter P_xyl/tet_	This study

### Generation of GBS mutant strains

In-frame deletion mutants of *ypmS* and SAK0604 in A909 and *ypmS* in AR1519 were created by allelic exchange using a temperature-sensitive shuttle vector with a counterselectable sucrose sensitivity cassette, pMBSacB [45]. A total of 800 bp of homology upstream and downstream of the target genes were cloned into pMBSacB, cut with XhoI and NotI, via Gibson assembly and transformed into 5-α competent *E. coli* (New England Biolabs). Because the CDSs of SAK0603, SAK0604 and *ypmS* overlap, the upstream arm for SAK0604 began at the stop codon of SAK0603, the downstream arm began at the start codon of *ypmS* and the upstream arm for *ypmS* began at the stop codon of SAK0604. This aimed to preserve the expression of genes surrounding the one targeted for deletion. The resulting plasmids were transformed into electrocompetent GBS strains A909 and AR1519. Single-cross plasmid insertions were selected for by growing the strains with erythromycin at 37 °C. Then double-cross plasmid excisions were selected for by growing the strains at 28 °C with no antibiotic selection. Lastly, strains were grown in sucrose to select for double-cross mutants which had completely removed the plasmid. Deletion mutants were confirmed via PCR and sequencing of the targeted deletion site.

The in-frame deletion mutant of *cpsE* in AR1519 was created by CRISPR/Cas12a genome editing using pGBSedit [46]. The Cas12a-compatible protospacer was designed using CRISPick [47] and ordered as forward and reverse oligos from Integrated DNA Technologies. The protospacer oligos were phosphorylated and cloned into pGBSedit, cut with Esp3I, using T4 ligase. The ligation product was transformed into 5-α competent *E. coli*. Then, 800 bp of homology up- and downstream of the target gene were cloned into the new plasmid cut with XhoI, via Gibson assembly. This plasmid was transformed into 5-α competent *E. coli* for storage and then transformed into electrocompetent GBS strain AR1519. The next day, transformants were used to start cultures in TS broth with 5 µg ml^−1^ of erythromycin and grown for 6–8 h. To induce Cas12a, 150 µl of the culture was mixed with 6 µl of 2 mg ml^−1^ aTC and plated on TS plates with 5 µg ml^−1^ of erythromycin and allowed to grow overnight, protected from light. Colonies were patched to a new TS erythromycin plate, and colony PCR was performed to identify successfully edited genomes. Lastly, mutants were cured of the pGBSedit plasmid, and their genotypes were confirmed via PCR and sequencing of the targeted deletion site.

To complement Δ*ypmS*, a new plasmid, pREGtet::*ypmS,* was created. First pREG696 [48] was digested with Smal and PstI to remove the spectinomycin resistance cassette. Then the erythromycin resistance cassette plus its promoter was PCR amplified from pOri23 [49], and the two pieces were Gibson assembled. The resulting plasmid, pREG696Erm, was transformed into electrocompetent Δ*ypmS* to be used as the empty vector control. To make pREGtet::*ypmS,* pREG696Erm was digested with BamHI and XbaI, *ypmS* was PCR amplified from A909, and the P_xyl/tet_ inducible promoter and TetR repressor were PCR amplified from pGBSedit. All three pieces were Gibson assembled and transformed into A909Δ*ypmS* and AR1519Δ*ypmS*. All primer and gene block sequences used in mutagenesis are listed in Table S1.

### Immunoblot

An individual colony for each GBS isolate was patched onto a fresh TS agar plate and incubated overnight at 37 °C. The next morning, a nitrocellulose membrane was applied to each plate and lifted to transfer patches. Membranes were blocked in 3% BSA in PBS (blocking solution) overnight at 4 °C. Membranes were transferred to blocking solution containing serotype-specific *Streptococcus* Group B serum (SSI Diagnostica) and incubated for 30 min with shaking at room temperature. Concentrations of each serotype-specific antibody used were as follows: Ia, 1 : 4,000; 1b, 1 : 4,000; II, 1 : 4,000; III, 1 : 20,000; IV, 1 : 25,000; V, 1 : 20,000; VI, 1 : 12,000; VII, 1 : 20,000; VIII, 1 : 20,000; and IX, 1 : 50,000. Blots were then washed thrice in PBS with shaking at room temperature for 10 min per wash. Blots were transferred to blocking solution containing the secondary antibody goat anti-rabbit IgG-horseradish peroxidase (HRP) (Invitrogen), diluted 1 : 1,000, and incubated for 2 h with shaking at room temperature. Blots were again washed thrice with PBS and then stained using a 3,3′-diaminobenzidine tetrahydrochloride substrate kit (Abcam). Clinical isolates were determined to be positive for the shedding phenotype by observing a ‘halo’ around the patch corresponding to capsule recognized by the antibody separate from the bacteria itself.

### Transposon screen

We performed a transposon screen to identify genetic determinants of CPS shedding. We utilized a previously created, indexed transposon library [34, 50] containing 1,920 individual mutants with unique transposon insertions throughout the A909 genome. Frozen stocks of each mutant were used to inoculate TS broth, which was incubated overnight under stationary conditions at 37 °C. The next day, 10 µl of each culture was spotted onto TS agar, and plates were incubated overnight at 37 °C. In the morning, spots were lifted from the plate with a nitrocellulose membrane. Membranes were immunoblotted with *Streptococcus* Group B type Ia serum (SSI Diagnostica) as described above. Transposon insertion mutants resulting in reduced or absent halos on the immunoblots were identified. Candidate non-shedding mutants were further screened using dot blots and ELLAs (described below) to differentiate mutants that were deficient in capsule production from mutants that retained surface-associated capsules and were specifically deficient in shedding.

### Dot blot

Dot blots were used to detect relative amounts of CPS present in the supernatant. GBS strains were grown overnight in TS broth with appropriate antibiotic supplementation under stationary conditions at 37 °C. In the morning, strains were subcultured and grown to an OD_600_ of 0.6. Cultures were then centrifuged, and the supernatant was sterilized using a 0.20 µm syringe filter to remove any bacteria. The supernatant was serially diluted 1 : 2, and 2 µl of each dilution was spotted in descending order onto a nitrocellulose membrane. Membranes were allowed to dry and then were blocked overnight at 4 °C in PBS with 3% BSA. The next day, membranes were immunoblotted with *Streptococcus* Group B type Ia serum as described in the immunoblot protocol above.

### Enzyme-linked lectin assay

As previously described, a whole-cell ELLA was used to compare relative amounts of surface-associated capsule on each strain [14]. An α2,3-linked sialic acid-specific lectin, *Maackia amurensis* lectin I (MAL I) was used to detect capsule. GBS strains were grown overnight in TS broth with appropriate antibiotic supplementation, stationary at 37 °C. In the morning, strains were subcultured and grown to an OD_600_ of 0.6. Strains were previously plated at this OD_600_ to confirm equivalent c.f.u. counts. Cultures were then centrifuged, and the pellet was resuspended in the same volume of coating buffer (0.087 M NaHCO_3_ and 0.015 M Na_2_CO_3_ in sterile water, pH 9.5). Suspensions were diluted 1 : 10 in coating buffer, and 100 µl of each strain was added to the wells of a column in an ELISA plate. The plate was incubated overnight at 37 °C.

The following day, any liquid left in the wells was removed, and wells were fixed with 50 µl of 100% methanol for 20 min at 37 °C. Excess methanol was removed, and wells were left to dry for another 20 min. Wells were then washed with 200 µl of PBS for 15 min at room temperature. Samples were blocked with 200 µl of 1% BSA in PBS for 4 h, covered, at 37 °C. After blocking, 50 µl of 10 µg ml^−1^ MAL I (Vector Laboratories) in 1% BSA, 0.05% Tween 20 in PBS (PBSAT) was added to the wells and incubated for 1 h, covered, at room temperature. Wells were subsequently washed three times with 200 µl of PBSAT for 5 min each. Streptavidin-HRP (Pierce) was diluted 1 : 20,000 in PBSAT, and 50 µl was added to each well and incubated for 15 min at 37 °C. Wells were again washed three times with 200 µl of PBSAT for 5 min each. A total of 100 µl of 3,3′,5,5′-tetramethylbenzidine-ELISA substrate solution (Pierce) was added to each well and incubated for 10 min at room temperature away from light. The reaction was stopped by adding 50 µl of 1 N H_2_SO_4_. Absorbance was read at 450 nm in a plate reader.

### Murine vaginal co-colonization model

All experimental procedures were reviewed and approved by the NYU Langone Institutional Animal Care and Use Committee (protocol IA16-01356). Six- to eight-week-old female C57BL/6 J mice were purchased from Jackson Laboratories. As previously described [14, 33, 51], the animals’ oestrous cycles were synchronized by subcutaneously injecting 10 µg of water-soluble 17β-oestradiol (Sigma-Aldrich) 48 and 24 h before colonization. Wild-type A909 and Δ*ypmS* strains were grown overnight. In the morning, the ODs of the two cultures were normalized to each other. Cultures were then centrifuged and resuspended in a 1 : 1 mixture of the two strains in PBS, which was subsequently mixed 1 : 1 with sterile 10% gelatine. The final concentration was 1×10⁹ c.f.u. ml^−1^. Mice were colonized under anaesthesia with 3–5% isoflurane (Baxter). Fifty microlitres of the GBS mixture (total inoculum 5×10⁷ c.f.u.) were administered intravaginally using a sterile pipette. Post-inoculation, mice were housed separately and swabbed on days 2, 4, 6, 8, 10 and 12 post-colonization. Vaginal swabs were vigorously stirred in 300 µl of sterile PBS. The swab solution was serially diluted and plated for c.f.u. enumeration. The dilutions were also spread on CHROMagar StrepB plates (CHROMagar) to generate single colonies and identify GBS among other vaginal microbes. Individual colonies from these plates were patched onto TS agar. Colony PCR was performed on 40 patches per mouse, per day to differentiate wild-type A909 from Δ*ypmS* colonies and subsequently calculate competitive index. Colony PCR was performed with SAK0605_check_fwd and SAK0605_check_rev primers (Table S1). Competitive index was calculated as


$$\frac{CFU\;wild-type\;recovered\;÷CFU\;wild-type\;inoculated}{CFU\;\Delta ypmS\;recovered\;÷CFU\;\Delta ypmS\;inoculated}=competitive\;index.$$

### Murine gastrointestinal co-colonization model

All experimental procedures were reviewed and approved by the NYU Langone Institutional Animal Care and Use Committee (protocol IA16-01356). Adult male and female C57BL/6 J mice (8–12 weeks) were purchased from Jackson Laboratories. As previously described [12, 52], mice were mated and dams were monitored for the birth of litters. Wild-type A909 and Δ*ypmS* strains were grown overnight. On the experimental day, the ODs of the two cultures were normalized to each other. Cultures were centrifuged and resuspended in a 1 : 1 mixture of the two strains in PBS. The final concentration was 1×10^9^ c.f.u. ml^−1^. Pups, age 12–14 days, were orally fed 50 µl of the GBS mixture using sterile feeding tubes and continued to be housed with their biological, non-colonized dam. Animals were monitored for signs of illness and mortality. At days 3, 7 and 14 post-infection animals were euthanized. One litter was used per time point. The GI tract was collected and divided into small intestine, caecum and large intestine sections. Each section was homogenized by bead-beating. Serial dilutions were spread plated on CHROMagar to generate single colonies and distinguish GBS from other microbes. Individual colonies from these plates were patched onto TS agar. Colony PCR was performed on 40 patches per mouse, per organ to differentiate wild-type A909 from Δ*ypmS* colonies and subsequently calculate competitive index using the same formula as above.

### Cell culture

HL60 cells (ATCC CCL-240) were grown in Roswell Park Memorial Institute (RPMI) 1640 with l-glutamine (Gibco) supplemented with 20% heat-inactivated FBS and penicillin–streptomycin at 37 °C and 5% CO_2_.

### Opsonophagocytic killing assay

OPK assays were performed as described by Nahm and Burton [53] with modifications. HL60 cells were differentiated with 0.8% *N*,*N*-dimethylformamide in RPMI 1640 and 20% heat-inactivated FBS at a density of 4×10^5^ cells ml^−1^. On day 5 post-differentiation, cells were harvested and resuspended in Hanks’ balanced salts solution with calcium and magnesium, 10% heat-inactivated FBS and 0.1% gelatine (Opsonization Buffer B [OBB]) to a final concentration of 1×10^7^ cells ml^−1^. For the standard OPK assay, overnight cultures of each strain being tested were subcultured and grown to an OD_600_ of 0.6. They were then diluted to a final concentration of 1×10^5^ c.f.u. ml^−1^ in OBB. Ninety-six-well plates were prepared with 20 µl of 1 : 3 serial dilutions of *Streptococcus* Group B type Ia serum, ranging from 1 : 300 to 1 : 218,700, diluted in OBB. Ten microlitres of bacteria were added to each dilution and to a well with OBB containing no antibody. The 96-well plate was then incubated at room temperature, shaking for 30 min. Baby rabbit complement was mixed 1 : 5 with the HL60 cells and 50 µl of this mixture was added to all wells. The plate was then incubated for 45 min at 37 °C in a 5% CO_2_ incubator. To stop the phagocytosis process, plates were placed on ice for 20 min. Five microlitres from each well was spotted onto square TS plates in triplicate and tilted sideways to spread out colonies for counting. The next day, CFUs were counted and percent survival was calculated as


$$\frac{median\;CFU\;with\;antibody}{median\;CFU\;without\;antibody}\;x\;100=\%\;survival.$$

For the OPK assays performed in the presence of supernatant, supernatants were prepared as described in the dot blot assay and then frozen at −20 °C until needed. Modifications to the OPK assay were as follows. The strain tested in the presence of all supernatants was the empty vector strain Δ*ypmS*+pREG696 Erm. *Streptococcus* Group B type Ia serum was diluted 1 : 2 from a concentration of 1 : 200 to 1 : 204,800 for the control curve or 1 : 800 for determining the effect of supernatant on survival. Antibody and Δ*ypmS*+pREG696 Erm were all diluted in supernatant instead of OBB for the initial incubation period.

### Statistical analyses

Statistics were calculated using GraphPad Prism 9 Software. ELLA absorbance and percent survival for the OPK assay were all compared using a one-way ANOVA with Tukey’s multiple comparisons test. A χ^2^ test was performed to compare frequency of shedding and non-shedding phenotypes between invasive and non-invasive clinical isolates. Significance was denoted as ns=no significance, *P<0.05, **P<0.01, ***P<0.001 and ****P<0.0001.

## Supplementary material

10.1099/mic.0.001763 Supplementary Material 1.

## Abbreviations

AP: antimicrobial peptide
aTC: anhydrotetracycline
CI: Confidence Interval
CPS: capsular polysaccharide
ELLA: enzyme-linked lectin assay
GBS: Group B *Streptococcus*
GI: gastrointestinal
HRP: horseradish peroxidase
LOD: late-onset disease
MAL I: *Maackia amurensis* lectin I
NYU: New York University
OBB: Opsonization Buffer B
OPK: opsonophagocytic killing
PBSAT: 1% BSA, 0.05% Tween 20 in phosphate-buffered saline
PCV13: 13-valent pneumococcal conjugate vaccine
RPMI: Roswell Park Memorial Institute
TS: tryptic soy
