# Supplementary material for: ypmS promotes capsular polysaccharide shedding and in vivo fitness of Group B Streptococcus
Source: Microbiology (Reading). 2026 Sep 2;172(9):001763. doi: 10.1099/mic.0.001763 (PMC13537807; doi:10.1099/mic.0.001763)
Supplement: Supplementary file 1 [file mic-172-01763-s001.pdf]

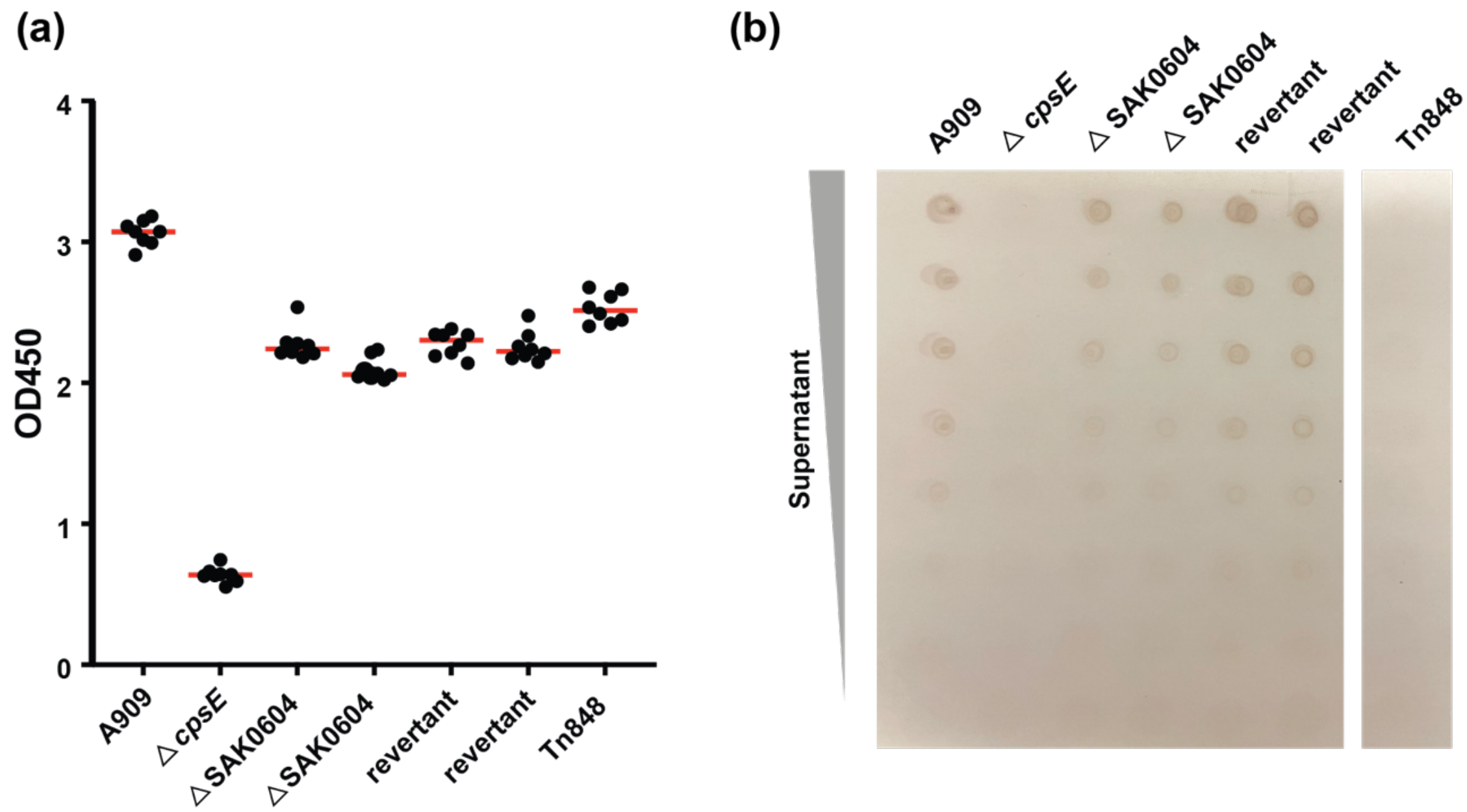

Supplemental Figure 1. Screening Tn848 and ΔSAK0604 for non-shedding phenotype. (a) Whole cell-ELLA using MAL I to assess surface-associated CPS. ANOVA with Tukey's multiple comparisons test was used to assess significance. A909 had significantly more and Δ*cpsE* had significantly less surface-associated capsule than all other strains ($p < 0.0001$). Among the mutants some small but statistically significant differences were detected. Tn848 had significantly more capsule than the ΔSAK0604 and revertant strains ($p < 0.0001$). The first ΔSAK0604 mutant was not significantly different than either revertant, while the second ΔSAK0604 mutant was significantly different with p-values of $p < 0.01$ and $p < 0.05$ in comparison with the two revertant strains respectively. (b) Dot blot of 1:2 dilutions of supernatant using a serotype Ia-specific antibody to detect shed CPS. Data from (a) and (b) were generated from the same culture grown to and OD_{600} of 0.6 and split into a (a) pellet fraction and a (b) supernatant faction. Duplicate ΔSAK0604 and wild-type revertant strains represent unique mutants created during the same mutagenesis process.

Supplemental Table 1. Primers used in this study

Primer / gBlock	Sequence (5'to 3')	Description
SAK0605_upstream_fwd	attgggtaccgggcccccccTGGTAAGTCTTCTTTTATCTTTTG	Amplifies upstream homology arm for Δ*ypmS* mutagenesis cassette from A909 to be Gibson assembled into pMBSacB digested with XhoI and NotI
SAK0605_upstream_rev	taacgtcattTTACGGCCAGTTTTTTCTTG	
SAK0605_downstream_fwd	ctggccgtaaAATGACGTTAAATACTTGACC	Amplifies downstream homology arm for Δ*ypmS* mutagenesis cassette from A909 to be Gibson assembled into pMBSacB digested with XhoI and NotI
SAK0605_downstream_rev	tggagctccaccgcggtggcTAGAATTCATAAATGAGCTAGAAAAG	
SAK0604_upstream_fwd	attgggtaccgggcccccccCCATTGAACCAGAACTTATTAAAG	Amplifies upstream homology arm for ΔSAK0604 mutagenesis cassette from A909 to be Gibson assembled into pMBSacB digested with XhoI and NotI
SAK0604_upstream_rev	cttgtttcatTTATTCATAACGAACCATAACAG	
SAK0604_downstream_fwd	ttatgaataaATGAAACAAGAAAAAACTGGC	Amplifies downstream homology arm for ΔSAK0604 mutagenesis cassette from A909 to be Gibson assembled into pMBSacB digested with XhoI and NotI
SAK0604_downstream_rev	tggagctccaccgcggtggcGCTGCAAAAACTGCATCTAC	
SAK0605_check_fwd	GTGTGGGCGATACAACCTCT	Binds wild-type A909 gDNA outside of the Δ*ypmS* mutagenesis cassette for confirmation of deletion genotype
SAK0605_check_rev	CAAACGAGCGGCTTGAACAA	
SAK0604_check_fwd	CATCACGCCAGGGAGCTAAT	Binds wild-type A909 gDNA outside of the ΔSAK0604 mutagenesis cassette for confirmation of deletion genotype
SAK0604_check_rev	ACCTTTACGAGCTGCACGTT	
pMBSacB_check_fwd	TGCAAGGCGATTAAGTTGGGTAA	Binds pMBSacB outside of XhoI and NotI cut sites for confirmation of mutagenesis cassette insertion
pMBSacB_check_rev	GCGGATAACAATTTCACACAGGAAA	
1519_ypmS_upstream_fwd	attgggtaccgggcccccccTGGTAAGTCTTCTTTTATCTTTTG	Amplifies upstream homology arm for Δ*ypmS* mutagenesis cassette from AR1519 to be Gibson assembled into pMBSacB digested with XhoI and NotI
1519_ypmS_upstream_rev	taacgtcattTTACGGCCAGTTTTTTTTTG	
1519_ypmS_downstream_fwd	ctggccgtaaAATGACGTTAAATACTTGACC	Amplifies downstream homology arm for Δ*ypmS* mutagenesis cassette from AR1519 to be Gibson assembled into pMBSacB digested with XhoI and NotI
1519_ypmS_downstream_rev	tggagctccaccgcggtggcAAACCTTGTTTGCTCTTTTATATTG	
1519_ypmS_check_fwd	CCGCCTTAGCTAACGTCCAT	Binds wild-type AR1519 gDNA outside of the Δ*ypmS* mutagenesis cassette for confirmation of deletion genotype
1519_ypmS_check_rev	CCGATTGTTAAAGGTCGCGG	

Primer / gBlock	Sequence (5'to 3')	Description
1519_cpsE_upstream_fwd<br>1519_cpsE_upstream_rev	aatttctactcttgtagatcTGTTAATACTGAAGAAGATATTGAAAAG<br>aaccttacctTACAAGAGCCCCTTACTTC	Amplifies upstream homology arm for Δ*cpsE* mutagenesis cassette from AR1519 to be Gibson assembled into pGBSedit digested with XhoI
1519_cpsE_downstream_fwd<br>1519_cpsE_downstream_rev	ggctcttgtaAGGTAAGGTTTGAAAGGAATATAATG<br>agcttgcatgtctgcaggccTATTAACAAAGTCCACCTG	Amplifies downstream homology arm for Δ*cpsE* mutagenesis cassette from AR1519 to be Gibson assembled into pGBSedit digested with XhoI
sgRNA_cpsE_fwd<br>sgRNA_cpsE_rev	TAGATGATATCACGGGTGCTATTATAGGA<br>AAATTCCTATAATAGCACCCGTGATATCA	Oligos designed for CRISPR/Cas12a targeting of *cpsE*. Annealed and ligated with pGBSedit cut with Esp3I
1519_cpsE_check_fwd<br>1519_cpsE_check_rev	CGGGTTTATTGTTGGTGCAGG<br>GTTTCCCTCGCCTAAACCCA	Binds wild-type AR1519 gDNA outside of the Δ*cpsE* mutagenesis cassette for confirmation of deletion genotype
oJC270<br>oJC271	AAAAAGATGCCAGTGTGCTG<br>CCAGTGCCAAGCTTGCATGT	Binds pGBSedit outside of XhoI cut site for confirmation of mutagenesis cassette insertion
crRNA_screen_rev	GCTATCTTCGTCATAGTTACCT	Binds pGBSedit outside of Esp3I cut site. To be used with sgRNA_cpsE_fwd for confirmation of protospacer insertion
pREG696_ErmR_fwd<br>pREG696_ErmR_rev	tgaccccgaagcaggctgcaGAGTGTGTTGATAGTGCAG<br>tggggatcggaattctccccggatccgtcgacctgtctagaAGGGTTTTCTCGATTCGTC	Amplifies the promoter and erythromycin resistance cassette from pOri23 to be Gibson assembled with pREG696 cut with SmaI and PstI
aTC+promoter_fwd<br>aTC+promoter_rev	cttgtttcatTAAAAACCTCCTTTACTGC<br>ggggatcggaattctccccgTTAAGACCCACTTTCACATTTAAG	Amplifies the $P_{xyl/tet}$ inducible promoter and TetR repressor from pGBSedit to be Gibson assembled with pREG696Erm cut with BamHI and XbaI
ypmS_complement_fwd<br>ypmS_complement_rev	gacgaatcgagaaaacccttTTAGGGCTTTTTCTTAAAAATATC<br>gaggtttttaATGAAACAAGAAAAAACTGG	Amplifies *ypmS* from A909 to be Gibson assembled with $P_{xyl/tet}$, TetR, and pREEG696Erm cut with BamHI and XbaI for complementation of Δ*ypmS*
